# Salivary pepsin as an independent predictor of treatment response for laryngopharyngeal reflux: prospective cohort study with multivariate analysis

**DOI:** 10.1038/s41598-023-50014-6

**Published:** 2023-12-21

**Authors:** Ji Min Yun, Ki Won Kim, Suji Kim, Yoon Kyoung So

**Affiliations:** 1 Department of Otorhinolaryngology-Head and Neck Surgery, Ilsan Paik Hospital, Inje University College of Medicine, 170 Juhwa-ro, Ilsanseo-gu, Goyang-si, Gyeonggi-do 10380 Republic of Korea; 2 Department of Otorhinolaryngology-Head and Neck Surgery, Severance Hospital, Yonsei University College of Medicine, Seoul, Republic of Korea

**Keywords:** Gastroenterology, Medical research

## Abstract

To analyze the predictive value of salivary pepsin for treatment outcomes in laryngopharyngeal reflux (LPR) using multivariate analysis that includes various associated factors. This prospective cohort study was conducted between August 2020 and August 2022. Patients with LPR who had symptoms lasting more than 1 month and a reflux symptom index (RSI) of 14 or higher were enrolled. The participants received a 2-month regimen of proton pump inhibitors (PPIs) treatment and lifestyle modification. Salivary pepsin was checked using fasting saliva before treatment. Salivary pepsin was detected more frequently in the good treatment response group (61.1%), compared to 14.3% in the poor response group. Similarly, patients with higher compliance to lifestyle modifications (> 90%) had a higher chance of a good response (91.7%) compared to those with lower compliance, who had a 53.8% chance of a good response. Other clinical factors have no significant association with treatment response. In multivariate analysis, both pretreatment salivary pepsin and higher compliance with lifestyle modification were found to be independent factors for treatment response (OR 14.457, CI 1.075 ~ 194.37 for both). This study found that positive salivary pepsin and strict lifestyle modification are independent predictors of treatment outcomes in LPR.

## Introduction

Laryngopharyngeal reflux (LPR) is a condition where gastric contents reflux beyond the laryngopharynx. The reflux directly irritates vocal folds and surrounding mucosa, causing globus and cough. Also, laryngopharyngeal tissue exposed for a long period to the refluxate can be damaged and show pathological changes^1^. LPR is an independent disease from gastroesophageal reflux disease (GERD), and its prevalence is increasing^2^. According to a survey in South Korea in 2000, 24 ~ 35% of patients in the otolaryngology department were found to have symptoms and findings related to LPR^3^. Patients with LPR exhibit a variety of symptoms, such as chronic cough, foreign body sensation, and voice changes^4^. If left untreated, LPR can result in severe complications such as chronic laryngitis, vocal cord granuloma, laryngeal stricture, and subglottic stenosis^5^.

Proton pump inhibitor (PPI) stands out as the commonly preferred first-line medication for addressing LPR^6^. In clinical practice, PPI is often prescribed empirically without confirmation of reflux^7,8^. However, LPR generally requires more prolonged treatment than GERD, and short-term PPI intake usually ends with treatment failure. The symptoms sometimes persist even with long-term PPI medication, leading to low satisfaction and destruction of rapport. In this context, the need for tests predicting responses to the empirical treatment has been raised. The salivary pepsin test has recently been studied as a non-invasive and practical tool for LPR treatment response.

Pepsin is a major component in the refluxate that damages the mucosa and can be detected in saliva. The salivary pepsin test uses collected saliva or sputum, which is non-invasive and easily performed by most patients^9,10^. Rapid lateral flow device (LFD) for salivary pepsin uses monoclonal antibodies against human pepsin A(isozymes 1, 3a, 3b, 3c)^11^. Peptest™ (RD Biomed, Hull, UK) is a commercially available LFD that detects pepsin in saliva within 15 min^12^. As well as the diagnostic accuracy of salivary pepsin for LPR, its predictive value for PPI treatment response is being studied using the Peptest device.

The response to LPR treatment can be influenced by various associating factors. Compliance with PPI medication and lifestyle modification are the typical factors. It refers to behaviors to reduce reflux, such as weight loss, keeping the head elevated while sleeping, and not eating within 2 to 4 h of sleep^13^. It also includes avoiding foods that cause or worsen the reflux, such as chocolate, high fat, carbonated drinks, spicy foods, tomatoes, caffeine, citrus fruits, and alcohol^7^. These behavioral and dietary modification has been reported to affect treatment response^14^. However, previous studies evaluating the treatment response with the salivary pepsin test did not consider these associating factors. Therefore, this study aims to analyze the predictive value of salivary pepsin for LPR treatment outcomes in multivariate analysis, including various associating factors such as compliance with lifestyle modifications.

## Method and materials

### Study population, setting, and design

This prospective cohort study was conducted on patients aged between 20 and 75 years who visited the otolaryngology department's outpatient clinic at a single university hospital from August 2020 to August 2022, complaining of symptoms of LPR lasting for more than a month. Only patients with a reflux symptom index (RSI) of 14 or higher were included, and those with conditions that could cause LPR-like symptoms were excluded. Exclusion criteria included acute upper respiratory infection (URI), acute or chronic sinusitis, allergic rhinitis, structural lesions in the pharynx, larynx, or oral cavity (such as tumors, lingual tonsil enlargement, epiglottic cysts, etc.), chronic respiratory diseases (such as asthma, chronic obstructive respiratory disease, tuberculosis, pneumonia, lung cancer, etc.), psychiatric illness history, or currently taking psychiatric medication. Pregnant females were also excluded. Patients who had used PPI, antacids, or mucosal protectants within the previous month were also excluded. The Institutional Review Board of Ilsan Paik Hospital approved this study (IRB File No. ISPAIK 2020-06-039-001), and it was conducted according to the Declaration of Helsinki. All subjects provided informed consent.

### Methods

During the first visit, patients were asked for their demographic and clinical information, including past medical history, medication history, smoking/alcohol history, sleep patterns, and caffeine intake. RSI and reflux finding score (RFS) were also recorded at this time. Based on RSI and endoscopic findings, oral ilaprazole (20 mg, once daily) was prescribed (20 mg, once daily). In addition, patients were educated about behavioral and dietary modifications that they should follow, which included the following instructions: (1) not lying down for 2 hours after a meal, (2) raising the pillow when sleeping, and (3) avoiding foods that cause reflux, such as alcohol, coffee, tea, soda, spicy foods, greasy foods, tomatoes, and onions.

Patients were given a checklist handbook to record their adherence to the above instructions and drug intake daily and were asked to submit it at the end of their treatment. The compliance rate was calculated by determining the percentage of days that each instruction was followed out of the total treatment days, and the compliance of behavioral/dietary modification was determined by taking the average compliance rates of the three instructions. Compliance with medication was calculated in the same manner. Compliance rates greater than or equal to 90% were considered high, while rates less than 90% were considered low.

RSI and RFS were re-evaluated 1 month and 2 months after the start of treatment, respectively. A good response to treatment was defined as a decrease in RSI score of more than 50% compared to the initial test after 2 months of treatment.

### Peptest

**Figure 1:**
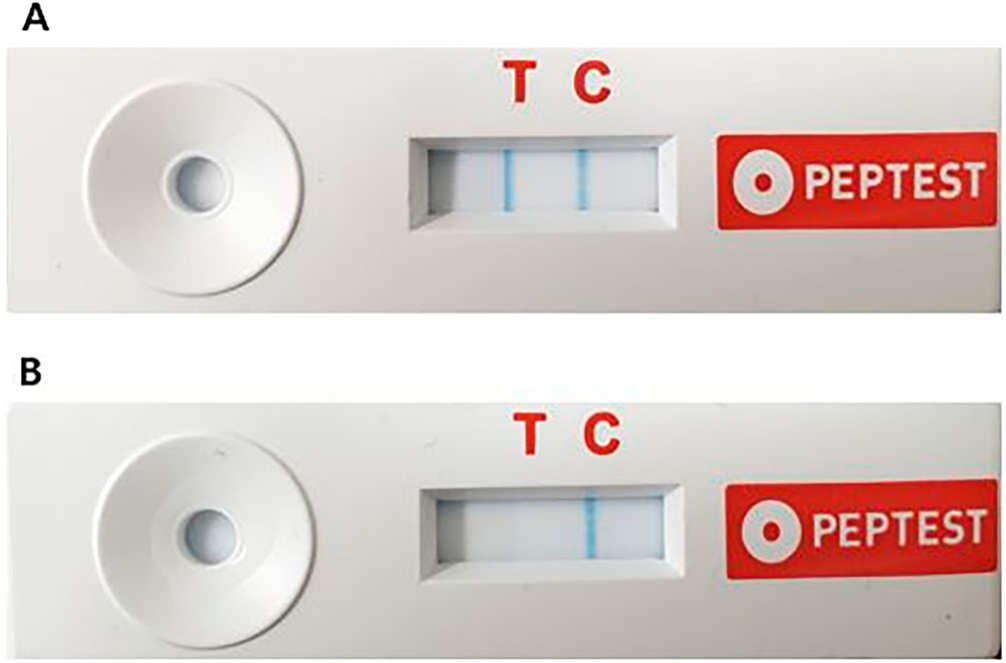
Pepsin lateral flow device for a saliva sample (**a**) A positive result showing blue bands in both test and control lines. (**b**) A negative result showing a band only in the control line.

**Figure 2:**
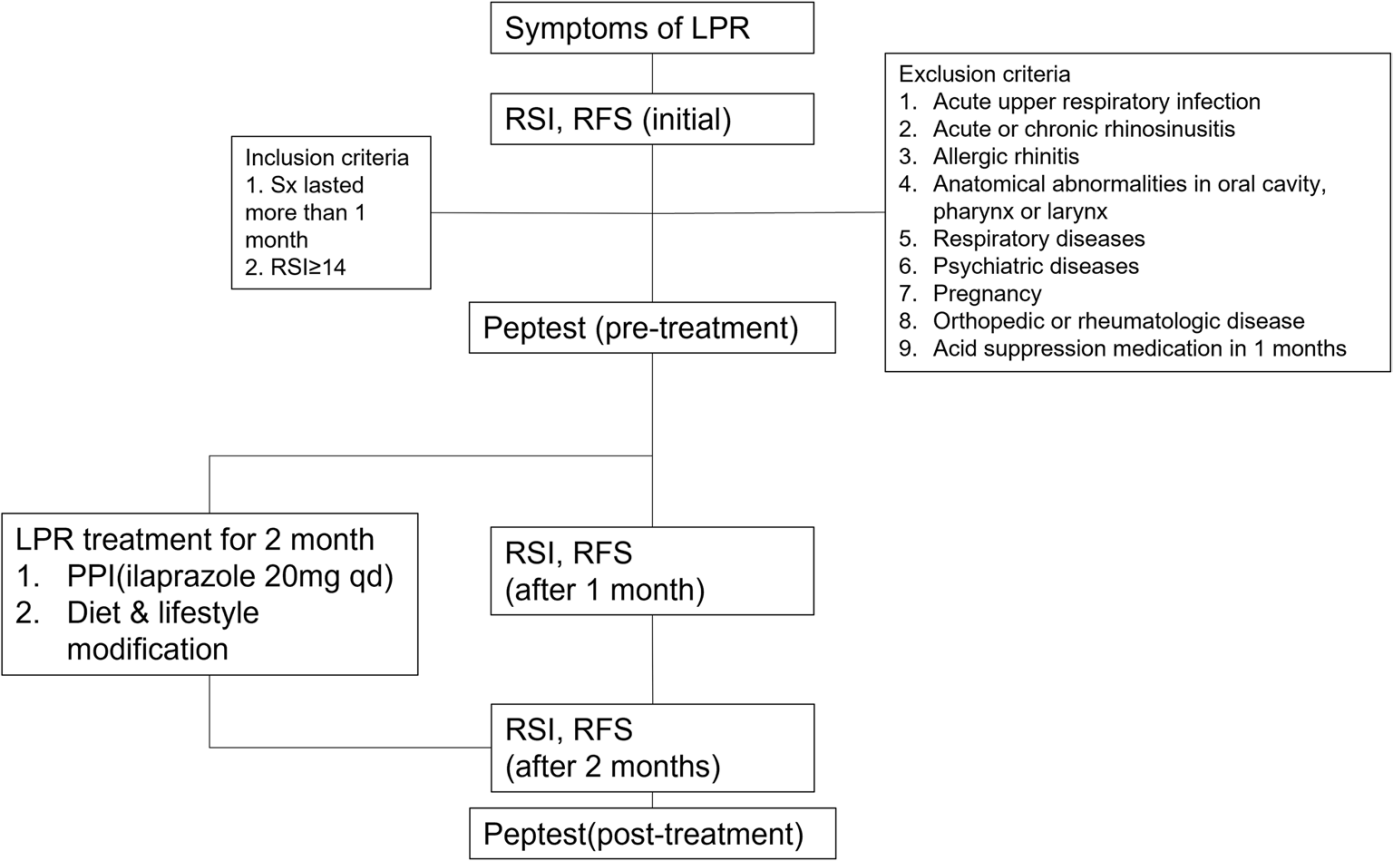
Flow diagram for study methods. LPR, Laryngopharyngeal reflux; RSI, Reflux symptom index; RFS, Reflux finding score; PPI, Proton pump inhibitor.

Patients were provided with a 30 ml universal sample collection tube for saliva collection and were instructed to collect saliva before and 2 months after starting treatment. Saliva was collected in the morning, immediately after waking up, and before consuming any food or water. After collection, the sample container was shaken to mix the saliva with the reagent (0.01 M citric acid 0.5 ml) inside. The container was first centrifuged at 4000 rpm for 5 min, and then 80 μL of the upper layer was transferred to a screw-top microtube containing 240 μL of migration buffer. The microtube was mixed with a vortex mixer for 10 s, and 80 μL of the mixed sample was administered to the circular well of the LFD. The test result was confirmed 15 min later, with a blue line appearing on the control line, indicating a valid test. A blue line on both the control and test lines indicated a positive result for pepsin in the saliva, with a concentration of 16 ng/mL or higher (Fig. 1). The pepsin concentration was quantitatively analyzed using the Peptest Cube (RD Biomed, Hull, UK), which measures the optical density of the test line through reflectance measurements. The research process of this study is depicted in Fig. 2. The main researcher who followed up with the patients was blinded to the result of the Peptest for each patient.

### Statistical analysis

Statistical analysis was performed using SPSS software version 23 (IBM Corporation, Armonk, NY, USA). The Shapiro–Wilk test was used to assess the normality of variables. Paired t-tests or Wilcoxon signed-rank tests were used to compare data before and after treatment, while independent t-tests or Mann–Whitney U tests were used to compare continuous variables between two groups. The Chi-square test was used to compare categorical variables between two groups. Logistic regression analysis was conducted to identify factors that may influence treatment response. All tests were two-sided, and *p* < 0.05 was considered statistically significant.

## Results

### Characteristics of the study population

Among the 31 patients who agreed to enroll, 25 completed all procedures. During the course of the study, three participants were excluded due to acute URI symptoms, and an additional three participants voluntarily dropped out due to the suspicion of COVID-19 in the context of the COVID-19 era. The mean age of the patients was 47.1 years (standard deviation [SD], 15.1 years). Sixteen of them were men (64%), and nine were women (36%). Of the 25 patients, 20 (80%) complained of globus, 8 (32%) of throat discomfort, 5 (25%) of voice change, and 2 (8%) of cough. The median value of symptom duration was 7 months (interquartile range [IQR], 1.5–54 months). The mean body mass index (BMI) was 24.8 kg/m^2^ (SD, 3.8). Three patients (12%) were current smokers, 16 (64%) were drinkers, and ten (40%) had sleep problems. Twelve patients (48%) reported having a dry mouth. The median compliance rate was 90.7% (IQR, 83.1–97.2%) with behavioral and dietary modifications, and 98.1% (IQR, 96.1–100.0%) with medication (Table 1).

**Table 1:** Patient’s characteristics.

Variables	Values
No. of patients	25
Age, yr	47.7 ± 15.1*
Sex	
Male (%)	16 (64.0)
Female (%)	9 (36.0)
Symptoms	
Globus (%)	20 (80.0)
Throat discomfort (%)	8 (32.0)
Voice change (%)	5 (25.0)
Cough (%)	2 (8.0)
Others (%)	1 (4.0)
Symptom duration, mo	7.0 (1.5 ~ 54.0)^†^
Smoking history	
Current smoker (%)	3 (12.0)
Current non-smoker (%)	22 (88.0)
Alcohol history	
Yes (%)	16 (64.0)
No (%)	9 (36.0)
BMI, kg/m^2^	24.8 ± 3.8*
Sleep problem	
Yes (%)	10 (40.0)
No (%)	15 (60.0)
Coffee drinker	
Yes (%)	17 (68.0)
No (%)	8 (32.0)
Dry mouth	
Yes (%)	12 (48.0)
No (%)	13 (52.0)
Pretreatment RSI	
Total	18.0 (16.0 ~ 24.5)^†^
Pretreatment RFS	
Total	12 (9 ~ 14)^†^
Pretreatment Peptest	
Positive (%)	12 (48.0)
Negative (%)	13 (52.0)
Pretreatment pepsin, ng/mL	16.0 (16.0 ~ 113.6)^†^

*Mean ± standard deviation, ^†^Median (Interquartile range).

RSI, Reflux symptoms index; RFS, Reflux finding score.

### Changes in RSI and RFS

Before treatment, the median value of RSI was 18 (IQR, 16.0–24.5), and the RSI categories with the highest scores were “clearing your throat” and “sensations of something sticking in your throat or a lump in your throat.” After 1 month of treatment, the RSI decreased significantly to 12.0 (IQR, 8.0–19.0), and 2 months after treatment, it decreased further to 8.0 (IQR, 4.5–11.5) (Wilcoxon signed rank test, all *p* < 0.001). Before treatment, the median RFS was 12.0 (IQR, 9.0–14.0), and the RFS categories with the highest score were RFS2 (ventricular obliteration) and RFS3 (erythema/hyperemia). After 1 month of treatment, RFS decreased significantly to 8.0 (IQR, 5.0–11.5), and after 2 months, it further decreased to 6.0 (IQR, 4.0–9.0) (Wilcoxon signed rank test, all *p* < 0.001 in Initial versus 1st FU, Initial vs. 2nd FU, and 1st FU vs. 2nd FU) (Table 2).

**Table 2:** The change of RSI and RFS during follow-up.

	RSI1	RSI2	RSI3	RSI4	RSI5	RSI6	RSI7	RSI8	RSI9	Total	P*
Initial	3	4	3	2	1	3	0	4	2	18.0(16.0 ~ 24.5)^†^	
1st FU	2	2	2	1	0	1	0	3	1	12.0(8.0 ~ 19.0)^†^	< 0.001**
2nd FU	1	1	1	0	0	0	0	1	0	8.0(4.5 ~ 11.5)^†^	< 0.001^‡^

	RFS1	RFS2	RFS3	RFS4	RFS5	RFS6	RFS7	RFS8	Total	P*	
Initial	0	4	4	1	1	1	0	2	12.0(9.0 ~ 14.0)^†^		
1st FU	0	2	2	1	1	1	0	0	8.0(5.0 ~ 11.5)^†^	< 0.001**	
2nd FU	0	2	2	1	1	1	0	0	6.0(4.0 ~ 9.0)^†^	< 0.001^‡^	

*Wilcoxon signed rank test, ^†^Values are expressed as a median(IQR for total scores), **Initial vs 1st FU, ^‡^Initial vs 2nd FU.

RSI, Reflux symptoms index; RFS, Reflux finding score; FU, follow-up.

RSI1: Hoarseness or a problem with your voice, RSI2: Clearing your throat, RSI3: Excess throat mucus or postnasal drip, RSI4: Difficulty in swallowing food, liquid, or pills, RSI5: Coughing after eating or after lying down, RSI6: Breathing difficulties or choking episodes, RSI7: Troublesome or annoying cough, RSI8: Sensation of something sticking in your throat or a lump in your throat, RSI9: Heartburn, chest pain, indigestion or stomach acid coming up.

RFS1: Subglottic edema (pseudosulcus), RFS2: Ventricular obliteration, RFS3: Erythema/hyperemia, RFS4: Vocal fold edema, RFS5: Diffuse laryngeal edema, RFS6: Posterior commissure hypertrophy, RFS7: Granuloma/granulation tissue, RFS8: Thick endolaryngeal mucus.

### Analysis of pepsin in saliva before and after treatment

Before treatment, 12 patients (48%) had a positive fasting Peptest, and 13 patients (52%) had a negative result. After treatment, 14 patients (56%) had a positive result, and 11 (44%) had a negative result. However, there was no significant difference in the positive rate before and after treatment (Chi-square test, *p* = 0.821). The median concentration of pepsin in saliva on the pretreatment Peptest was 16.0 ng/mL (IQR, 16.0–113.6), and the median concentration on the posttreatment Peptest was 49.2 ng/mL (IQR, 8.0–141.0), indicating no significant difference before and after treatment (Wilcoxon signed-rank test, *p* = 0.935).

### Comparison of the good response group and poor response group

The study analyzed factors that could affect the treatment response in patients with laryngopharyngeal reflux (LPR). The patients were divided into two groups: the good response group (n = 18) and the poor response group (n = 7), based on whether their Reflux Symptom Index (RSI) decreased by more than 50% after treatment compared to before treatment. The following factors were compared between the two groups: age, BMI, duration of symptoms, drinking, smoking, coffee intake, irregular sleep pattern, dry mouth, compliance with behavioral/dietary modifications, Peptest positivity before treatment, and pepsin concentration before treatment (Table 3). The median RSI before treatment was 17.5 (IQR, 16.0–26.5) in the good response group and 19.0 (IQR, 15.0–22.0) in the poor response group, showing no significant difference between the two groups (Mann–Whitney U test, *p* = 0.836). Including the initial RSI, there was no significant difference between the two groups in most associating factors.

**Table 3:** Comparison of clinical characteristics between treatment response groups.

Variable	Good response (n = 18)	Poor response (n = 7)	*P*
Age	50.39 ± 13.49	40.71 ± 17.91	0.181*
BMI	24.38 ± 3.85	25.94 ± 3.56	0.806*
Symptom duration (mo)	5.0 (1.0 ~ 42.0)	12.0 (7.0 ~ 60.0)	0.141^†^
Smoking	2 (11.1%)	1 (14.3%)	0.826^‡^
Alcohol	11 (61.1%)	5 (71.4%)	0.629^‡^
Coffee drinking	1.0 (0.0 ~ 3.0)	1.0 (0.0 ~ 2.0)	0.657^†^
Sleep problem	7 (38.9%)	3 (42.9%)	0.856^‡^
Dry mouth	9 (50.0%)	3 (42.9%)	0.748^‡^
Initial RSI	17.5 (16.0 ~ 26.5)	19.0 (15.0 ~ 22.0)	0.836^†^
Compliance with lifestyle modification (percent)	93.6 (89.1 ~ 97.5)	79.2 (72.2 ~ 89.9)	0.047^†^
Compliance with lifestyle modification (≥ 90%)	11 (61.1%)	1 (14.3%)	0.035^‡^
Compliance with medication (percent)	98.1 (96.0 ~ 98.4)	100.0 (96.4 ~ 100.0)	0.110^†^
Compliance with medication (≥ 90%)	16 (88.9%)	7 (100%)	0.358^‡^
Initial peptest (positive)	11 (61.1%)	1 (14.3%)	0.035^‡^
Initial peptest (concentration)	38.2 (16.0 ~ 146.9)	16.0 (16.0 ~ 16.0)	0.244^†^

*Independent *t*-test, ^†^Mann–Whitney U test, ^‡^Chi-square test.

Values are expressed as Mean ± SD for age, BMI and as Median (IQR) for symptom duration, coffee drinking, initial RSI, compliance with lifestyle modification (percent), compliance with medication, and initial Peptest (concentration). Otherwise, values are expressed as N (%).

RSI, Reflux symptom index.

**Figure 3:**
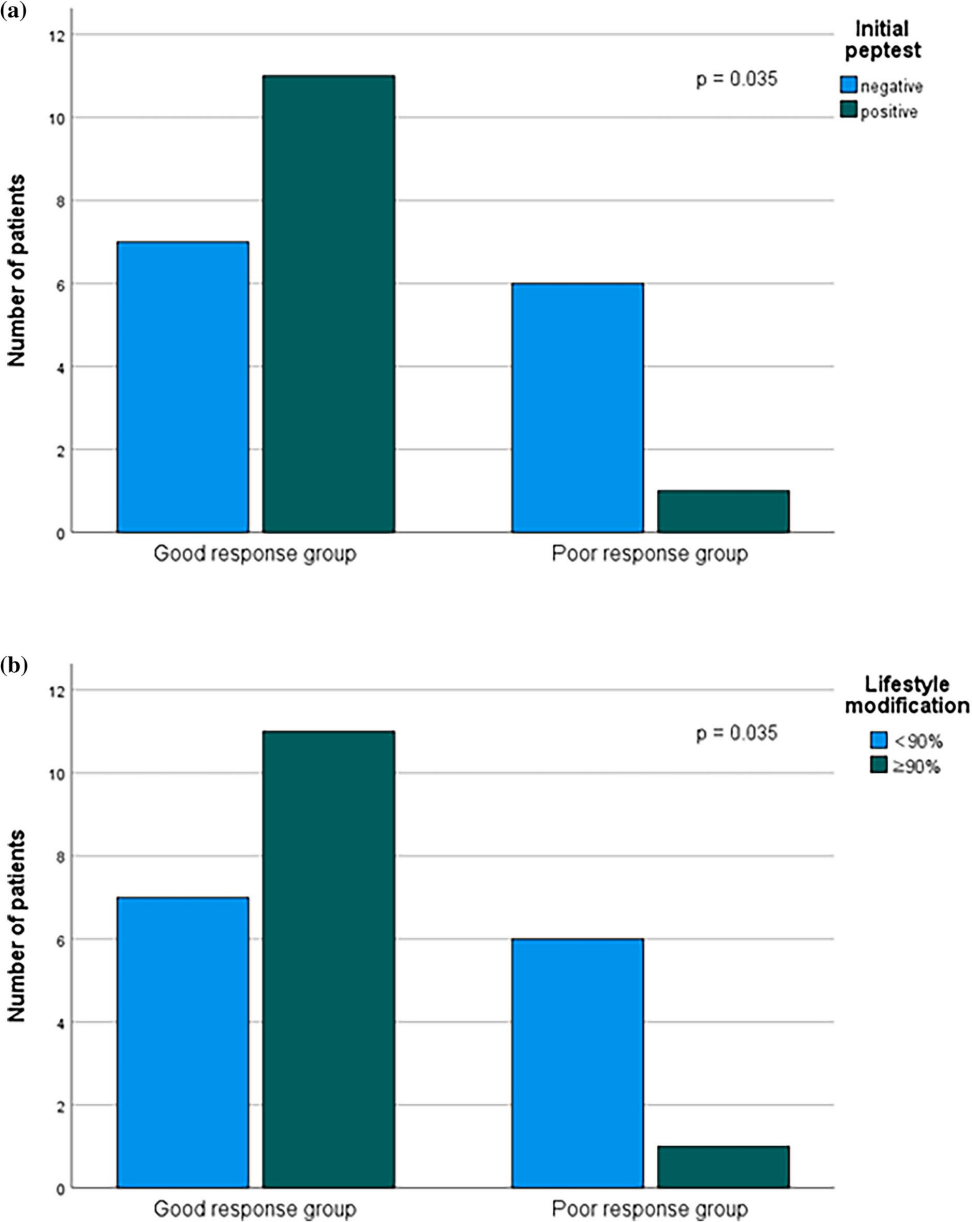
Comparison between the good response group and poor response group (**a**) The positive Peptest before treatment was more frequently observed in the good response group (Chi-square test, *p* = 0.035). (**b**) High compliance to lifestyle modification was more frequently observed in the good response group (Chi-square test, *p* = 0.035).

However, compliance with behavioral/dietary modifications was significantly higher in the good response group with a median value of 93.6% (IQR, 89.1–97.5) than 79.2% (IQR, 72.2–89.9) in the poor response group (Mann–Whitney U test, *p* = 0.047). When compliance was classified as high (≥ 90%) and low (< 90%), 11 out of 18 patients (61.1%) from the good response group and 1 out of 7 patients (14.3%) from the poor response group had high compliance (Chi-square test, *p* = 0.035). Peptest positivity before treatment was also significantly higher in the good response group. The positive rate was 61.1% in the good response group and 14.3% in the poor response group (Chi-square test, *p* = 0.035) (Fig. 3).

We conducted a multivariate logistic regression analysis of the factors affecting treatment response, including Peptest positivity before treatment and compliance with behavioral/dietary modifications, both of which showed statistical significance in univariate analysis, along with drinking history, which is generally considered an important factor for LPR. Alcohol history was not a significant factor for treatment response (*p* = 0.958). In the case of Peptest positivity before treatment and high compliance with behavioral/dietary modifications, the odds ratio for a good treatment response was 14.457 (95% CI 1.075–194.369, *p* = 0.044), respectively, indicating that these were significant factors for a good response (Table 4).

**Table 4:** Logistic regression analysis for treatment response.

Variable	OR	95% CI	*P*
Initial peptest (positive)	14.457	1.075 ~ 194.37	0.044
Lifestyle modification (≥ 90%)	14.457	1.075 ~ 194.37	0.044
Alcohol history	1.077	0.068 ~ 17.022	0.958

## Discussions

Pepsin is a proteolytic enzyme that is secreted by the gastric chief cells into the lumen of the stomach as an inactive form, pepsinogen. It is activated to pepsin in an acidic environment with a pH of 2.0 and is inactivated again at a pH of 6.0 or higher^15^. Pepsin may remain inactive when refluxed to the laryngopharynx, as its pH is 6.8. However, it can remain stable in the epithelium for at least 24 h, even at a pH of 7.0, and can be activated when acid reflux occurs later, resulting in its resumed proteolytic activity^16^. Furthermore, pepsin can contribute to the inflammatory response of laryngopharyngeal mucosa, even in a non-acidic environment. It can enter cells through endocytosis and be activated in the Golgi body, resulting in intracellular damage, or it can increase the expression of inflammatory cytokines in hypopharyngeal cells^17,18^.

Since pepsin can only be present in the laryngopharynx via reflux, the presence of pepsin in saliva can indicate reflux. Several studies have investigated the diagnostic value of salivary pepsin in laryngopharyngeal reflux (LPR). For example, Wang et al. detected pepsin in saliva in LPR patients and a control group using Enzyme-Linked Immunosorbent Assay (ELISA), and found that the average pepsin concentration in LPR patients was significantly higher (199.59 ng/mL) than in the control group (0.36 ng/mL)^19^. Weitzendorfer et al. reported a specificity of 86.2% and a sensitivity of 41.5% for diagnosing LPR using salivary pepsin with an optimal cutoff value of 216 ng/mL^20^. Similarly, Wang et al. reported a specificity of 84.4% and a positive predictive value of 79.2% for strong positive Peptest results of 250 ng/mL or more^21^. Another study suggested that Peptest had a higher diagnostic value than the combined tests of RSI and RFS^22^.

In contrast to these findings, some studies suggested that salivary pepsin cannot be a reliable tool for diagnosing LPR. Bobin et al. reported no correlation between reflux occurrences confirmed by 24-h MII-pH monitoring and the concentration of pepsin in saliva measured during reflux^23^. Yadlapati et al. claimed that the current threshold of Peptest (16 ng/mL) could not differentiate between patients and normal groups^24^. According to the recently published meta-analyses, the diagnostic value of salivary pepsin varies depending on the study design^25,26^. Inconsistencies in sample size, diagnostic criteria of LPR, test method (ELISA/Western blot/Peptest), sampling timing, and pepsin diagnostic threshold among different studies necessitate further research to have a reliable conclusion on this.

The treatment of LPR consists of medications that reducethe acidity (pH) of gastric contents, such as PPIs, H2-blockers, and antacids, as well as modifications of behavioral/dietary habits that cause or worsen reflux. PPI, a first-line medicine for LPR, reduces gastric acid secretion, reducingthe acidity of gastric contents^27^. It also inhibits the conversion of pepsinogen to pepsin, thereby preventing mucosal damage caused by pepsin^28^. However, in cases of LPR caused by mild or non-acidic reflux, 50 to 74% of patients are refractory to PPI treatment^29,30^. Additionally, PPIs do not prevent reflux itself^31^. Therefore, although PPI treatment removes the acidity of reflux, the reflux phenomenon may persist, allowing pepsin or pepsinogen to continue to reflux into the pharynx. In this context, modifying behavioral/dietary habits to reduce reflux is necessary.

The response to treatment for LPR is relatively low compared to GERD, and there is significant inter-individual variation^8,30^. If the treatment outcome can be predicted before PPI treatment, it is possible to set a personalized treatment direction at an early stage. Recently, studies have attempted to predict the treatment outcome using Peptest. Lechien et al. performed Peptest on 124 patients diagnosed with LPR through 24-h MII pH monitoring. They confirmed that the improvement of symptoms and endoscopic findings was better in patients with a positive Peptest before treatment than in those with a negative Peptest^32^. Wang et al. conducted Peptest before treatment on 74 patients and measured the pepsin concentration. They found that the response to PPI treatment was the best in patients with a strong positive Peptest, and the positive predictive value was reported as 79.2%^21^.

In this study, it was found that while the RSI and RFS improved after treatment, there was no significant difference in the Peptest positive rate and concentration before and after treatment. Considering findings from prior studies suggesting that PPIs do not reduce pepsin secretion or reflux events, the level of refluxed pepsin might persist without significant change following PPI treatment alone. Additionally, the Peptest's inability to reflect pepsin activity might result in the detection of inactive pepsin in the pharynx due to the absence of the acidic conditions necessary for activation. On the other hand, behavioral and dietary modifications are acknowledged not only for reducing gastric acidity but also for addressing esophageal sphincter dysfunction and enhancing gastric/esophageal motility, consequently diminishing the frequency of reflux events^33–35^. Despite the reduction in reflux events resulting from behavioral/dietary modifications, a decrease in pepsin concentration may not consistently occur, influenced by various factors. These include the potential increase in pepsin concentration in gastric juice resulting from PPI use. In a previous study, PPIs were found to decrease the overall volume of gastric secretion without affecting gastric pepsin output, leading to an observed rise in pepsin concentration within the gastric secretion^27^. Subsequent research is needed to determine whether the concentration of refluxed pepsin in the pharynx decreases through behavioral/dietary modification treatment alone. The authors suggest that LPR treatment, including PPI medication and behavioral/dietary modification, reduces the acidity in the pharynx and minimizes reflux episodes. Despite the lack of change in the concentration of pepsin in the pharynx, this may still lead to the transformation of pepsin into an inactive state, thereby preventing mucosal damage caused by both pepsin and acid.

Since the concentration of salivary pepsin did not accurately reflect the improvement of LPR symptoms, Peptest has its limitations in assessing the severity of symptoms. Additionally, follow-up of Peptest after treatment is considered less effective.

However, this study confirmed the potential use of Peptest as a predictor of treatment response. Patients with a positive Peptest before treatment were significantly more likely to have a good treatment response compared to those with a negative Peptest (OR 14.457, 95% CI 1.075–194.37, *p* = 0.044). The authors hypothesized that active pepsin may play a significant role in the development of LPR in patients with high levels of pepsin refluxed into the pharynx. As a result, a decrease in pepsin activity following treatment may lead to symptom improvement. Conversely, patients with pepsin levels below a certain threshold may not respond to treatments that modify pepsin activity, suggesting that pepsin may not be the primary cause of their LPR. For patients with a negative Peptest, additional tests such as a detailed medical history, physical examination, intraluminal impedance/pH monitoring, and esophageal manometry may be necessary to determine the cause of LPR at an early stage.

In addition to Peptest results, compliance with behavioral/dietary modifications was found to influence treatment response. Patients with a compliance rate of 90% or higher were significantly more likely to respond to treatment than those with less than 90% compliance (OR 3.3, 95% CI 1.2–8.7, *p* = 0.023). Therefore, it is recommended to provide patients with sufficient education before starting treatment and to distribute self-checkup handbooks for daily compliance self-assessment, as attempted in this study. Through this handbook, physicians can analyze and correct the causes of treatment failure in refractory patients. Moreover, as compliance with behavioral and dietary modifications emerged as an independent factor in the multivariate analysis, there is a potential for LPR improvement through treatment solely based on behavioral/dietary modifications. Otherwise, factors known to be associated with LPR, such as obesity, smoking, alcohol consumption, caffeine intake, sleep pattern abnormalities, and dry mouth, did not affect treatment response in this study. The severity and duration of symptoms before treatment were also found to be unrelated to treatment response.

This study differs from previous studies that used Peptest to predict LPR treatment response in several ways. Peptest was performed before and after treatment to examine changes in salivary pepsin. Behavioral and dietary habits were modified in addition to PPI medication for treating LPR, and compliance was assessed to analyze its effect on treatment response. Lastly, the study evaluated whether various clinical factors, in addition to salivary pepsin, affected treatment response. Limitations of this study include the small sample size, and the diagnosis of LPR and assessment of treatment response were only based on RSI without objective tests such as 24-h multichannel intraluminal impedance-pH monitoring. However, this is the usual diagnostic approach in clinical practice and has been adopted in previous studies. In addition, our study exclusively incorporated PPIs for medication treatment, which may be less effective in cases of mild or non-acidic reflux, primarily due to the absence of an evaluation for reflux type. Further investigations employing personalized medication treatments are needed.

## Data Availability

The datasets generated during and/or analyzed during the current study are available from the corresponding author on reasonable request.
